# Maternal HIV-1 Env Vaccination for Systemic and Breast Milk Immunity To Prevent Oral SHIV Acquisition in Infant Macaques

**DOI:** 10.1128/mSphere.00505-17

**Published:** 2018-01-10

**Authors:** Joshua A. Eudailey, Maria L. Dennis, Morgan E. Parker, Bonnie L. Phillips, Tori N. Huffman, Camden P. Bay, Michael G. Hudgens, Roger W. Wiseman, Justin J. Pollara, Genevieve G. Fouda, Guido Ferrari, David J. Pickup, Pamela A. Kozlowski, Koen K. A. Van Rompay, Kristina De Paris, Sallie R. Permar

**Affiliations:** a Duke Human Vaccine Institute, Duke University Medical Center, Durham, North Carolina, USA; b Department of Microbiology and Immunology and Center for AIDS Research, School of Medicine, University of North Carolina at Chapel Hill, Chapel Hill, North Carolina, USA; c Department of Surgery, Duke University School of Medicine, Durham, North Carolina, USA; d Department of Biostatistics, Gillings School of Public Health, University of North Carolina at Chapel Hill, Chapel Hill, North Carolina, USA; e Department of Pathology and Laboratory Medicine, University of Wisconsin-Madison, Madison, Wisconsin, USA; f Department of Molecular Genetics and Microbiology, Duke University Medical Center, Durham, North Carolina, USA; g Department of Microbiology, Immunology and Parasitology, Louisiana State University Health Sciences Centre, New Orleans, Louisiana, USA; h California National Primate Research Center, University of California at Davis, Davis, California, USA; Icahn School of Medicine at Mount Sinai

**Keywords:** HIV-1, breast milk, maternal vaccination, oral challenge, placental transfer, transmission

## Abstract

Mother-to-child transmission (MTCT) of human immunodeficiency virus type 1 (HIV-1) contributes to an estimated 150,000 new infections annually. Maternal vaccination has proven safe and effective at mitigating the impact of other neonatal pathogens and is one avenue toward generating the potentially protective immune responses necessary to inhibit HIV-1 infection of infants through breastfeeding. In the present study, we tested the efficacy of a maternal vaccine regimen consisting of a modified vaccinia virus Ankara (MVA) 1086.C gp120 prime-combined intramuscular-intranasal gp120 boost administered during pregnancy and postpartum to confer passive protection on infant rhesus macaques against weekly oral exposure to subtype C simian-human immunodeficiency virus 1157ipd3N4 (SHIV1157ipd3N4) starting 6 weeks after birth. Despite eliciting a robust systemic envelope (Env)-specific IgG response, as well as durable milk IgA responses, the maternal vaccine did not have a discernible impact on infant oral SHIV acquisition. This study revealed considerable variation in vaccine-elicited IgG placental transfer and a swift decline of both Env-specific antibodies (Abs) and functional Ab responses in the infants prior to the first challenge, illustrating the importance of pregnancy immunization timing to elicit optimal systemic Ab levels at birth. Interestingly, the strongest correlation to the number of challenges required to infect the infants was the percentage of activated CD4^+^ T cells in the infant peripheral blood at the time of the first challenge. These findings suggest that, in addition to maternal immunization, interventions that limit the activation of target cells that contribute to susceptibility to oral HIV-1 acquisition independently of vaccination may be required to reduce infant HIV-1 acquisition via breastfeeding.

**IMPORTANCE** Without novel strategies to prevent mother-to-child HIV-1 transmission, more than 5% of HIV-1-exposed infants will continue to acquire HIV-1, most through breastfeeding. This study of rhesus macaque dam-and-infant pairs is the first preclinical study to investigate the protective role of transplacentally transferred HIV-1 vaccine-elicited antibodies and HIV-1 vaccine-elicited breast milk antibody responses in infant oral virus acquisition. It revealed highly variable placental transfer of potentially protective antibodies and emphasized the importance of pregnancy immunization timing to reach peak antibody levels prior to delivery. While there was no discernible impact of maternal immunization on late infant oral virus acquisition, we observed a strong correlation between the percentage of activated CD4^+^ T cells in infant peripheral blood and a reduced number of challenges to infection. This finding highlights an important consideration for future studies evaluating alternative strategies to further reduce the vertical HIV-1 transmission risk.

## Abstract (precis)

Without novel strategies to prevent mother-to-child HIV-1 transmission, more than 5% of HIV-1-exposed infants will continue to acquire HIV-1, most through breastfeeding. This study of rhesus macaque dam-and-infant pairs is the first preclinical study to investigate the protective role of transplacentally transferred HIV-1 vaccine-elicited antibodies and HIV-1 vaccine-elicited breast milk antibody responses in infant oral virus acquisition. It revealed highly variable placental transfer of potentially protective antibodies and emphasized the importance of pregnancy immunization timing to reach peak antibody levels prior to delivery. While there was no discernible impact of maternal immunization on late infant oral virus acquisition, we observed a strong correlation between the percentage of activated CD4^+^ T cells in infant peripheral blood and a reduced number of challenges to infection. This finding highlights an important consideration for future studies evaluating alternative strategies to further reduce the vertical HIV-1 transmission risk.

## INTRODUCTION

In 2015, an estimated 150,000 children were newly infected with human immunodeficiency virus type 1 (HIV-1) worldwide (1). While antiretroviral therapy (ART) can dramatically reduce the rate of mother-to-child transmission (MTCT) (2), in areas of high HIV-1 prevalence, there is poor access and adherence to ART throughout the breastfeeding period, which constitutes a major risk for HIV-1 transmission to the infant (3). Accordingly, as long as ART coverage cannot be improved, >5% of infants born to mothers living with HIV-1 will continue to acquire HIV-1, most of which will occur through breastfeeding (4).

One potential intervention to further reduce the rate of postpartum MTCT is maternal vaccination. Maternal immunization could enhance antiviral immune responses that neutralize or block viruses in the mother, as well as provide passive immunization to the infant through the transfer of antibodies (Abs) from mother to infant via the placenta and through breastfeeding (5). Maternal immunization is especially attractive because neonatal vaccines administered after birth do not start providing adequate protection until the infant is at least several months old (6). Maternal vaccination has been established as effective in alleviating the burden of neonatal illness and death due to pathogens such as influenza, pertussis, and tetanus (7–17). In addition to the efficacy of these maternal vaccines, multiple studies have demonstrated their safety and a lack of association with adverse birth outcomes (18–22).

Defining the maternal immune correlates of risk of vertical HIV-1 transmission can identify potential targets of vaccine-elicited immunity that will confer protection on the infant. We previously examined a pre-ART era U.S. cohort of nonbreastfeeding HIV-1-infected mothers from the Women and Infants Transmission Study (WITS) to define the immune correlates of risk of *in utero* and peripartum transmission (23). The analyses revealed that maternal systemic IgG binding to the third variable loop (V3) of the HIV-1 envelope (Env) was predictive of a reduced MTCT risk, confirming a previously identified association (24). In addition, neutralization of easy-to-neutralize (tier 1A) clade-matched HIV-1 variants, but not tier 2 difficult-to-neutralize variants, predicted reduced peripartum transmission. Importantly, these potentially protective responses were highly correlated and colinear, suggesting that V3-specific autologous neutralizing Abs in HIV-1-infected pregnant women may further reduce peripartum transmission of HIV-1. In fact, these types of V3-specific autologous virus-neutralizing Abs were isolated from a nontransmitting mother in the WITS cohort (23).

In another study, we identified an association between the magnitude of Env-specific IgA and secretory IgA (sIgA) in breast milk and a reduced transmission risk in a cohort of HIV-1-infected postnatally transmitting and nontransmitting mothers from the Malawi-based Breastfeeding Antiretrovirals and Nutrition Study (25). This observation was surprising, as sIgA is the predominant immunoglobulin isotype found in milk (26), yet milk HIV-1 Env-specific IgG responses are greater in magnitude and mediate most of the antiviral activity measured in breast milk (27–30). These findings suggest that mucosal Env-specific IgA may play a role in the setting of postnatal MTCT and merit further investigation to determine if a maternal vaccine capable of eliciting robust IgA responses in milk could provide protection against breast milk transmission.

The safety and immunogenicity of maternal HIV-1 Env vaccination to reduce MTCT have previously been evaluated in an early-phase clinical trial. A study conducted in the mid-1990s enrolled 26 HIV-1-infected pregnant U.S. women in a placebo-controlled study evaluating the safety and immunogenicity of an MN rgp120 subunit vaccine adjuvanted with aluminum hydroxide (alum) (31). Study subjects received an initial intramuscular (i.m.) injection of MN rgp120 or diluent at enrollment during the second trimester of pregnancy, followed by monthly injections until the end of pregnancy. Vaccination was well tolerated with no local or systemic reactions in the mothers and no adverse outcomes in the infants attributable to the vaccine. Because of the small size of the study, factors influencing transmission and vaccine efficacy were not assessed. Although there was limited evidence of boosting of heterologous virus-neutralizing responses and equivalent rates of transmission in vaccinated (3/17 [18%]) and unvaccinated (2/9 [22%]) mothers, this study demonstrated that an HIV-1 vaccine can be safely administered to HIV-1-infected pregnant women.

We previously evaluated an HIV-1 Env prime-boost maternal vaccine regimen in hormone-induced lactating rhesus monkeys (32). These animals were primed with modified vaccinia virus Ankara (MVA) expressing the gp140 Env gene of the clade C transmitted/founder (T/F) virus 1086.C (33) and subsequently boosted twice i.m. or intranasally (i.n.) with HIV-1 1086.C gp120 protein. Both systemic and mucosal immunization strategies elicited similar levels of HIV-1 Env-specific IgG in milk and plasma, but the mucosal immunization induced a higher magnitude Env-specific IgA response in milk. Additionally, vaccine-elicited Ab-dependent cell-mediated cytotoxicity (ADCC) responses, although present in the plasma of both i.m. and i.n. immunized monkeys, were present only in the milk of monkeys in the i.m. group, potentially because of IgA blocking. Interestingly, a combined i.m.-i.n. boost induced both high levels of Env-specific IgA and tier 1 neutralization in breast milk (34). Moreover, a large proportion of the vaccine-elicited memory B cell population in milk expressed IgA and isolated Env-specific breast milk monoclonal Abs (MAbs) exhibited diverse epitope specificities and multiple effector functions, including ADCC, tier 1 neutralization, infected cell binding, and inhibition of viral attachment to epithelial cells. Building on the establishment of this previous vaccine regimen that achieved both robust systemic IgG responses and milk IgA responses, this study aimed to test the immunogenicity of an HIV-1 Env maternal vaccine regimen in the setting of pregnancy and examine the role of the vaccine-elicited transplacentally transferred IgG and milk IgA in protection against oral virus transmission in the infant.

## RESULTS

### Maternal i.m. MVA gp120 prime–i.m.-i.n. gp120 boost immunization strategy proves safe and elicits high levels of plasma IgG and milk IgA.

**FIG 1:**
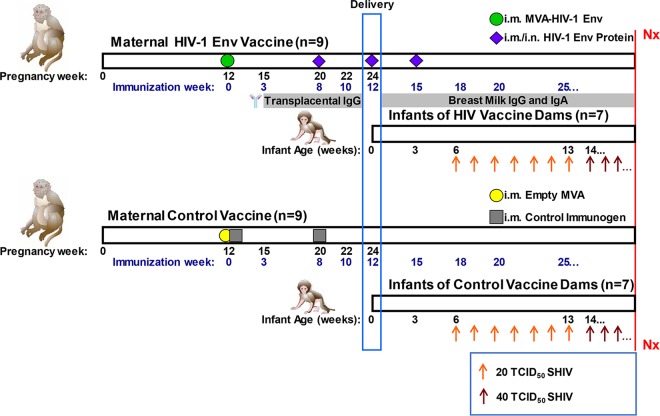
i.m. MVA gp120 prime–i.m.-i.n. gp120 boost immunization schedule. Nine rhesus monkey dams in the treatment group (top panel) were primed by immunization with an MVA expressing 1086.C gp120 at 12 weeks of gestation, followed by three successive i.m.-i.n. boosts with 1086.C in STR8S-C adjuvant (i.m.) or in R848 (i.n.). Nine rhesus monkey dams in the control group (bottom panel) received an empty MVA vector plus a control immunogen at 12 weeks of gestation, followed by a single boost. Gestation of macaques is approximately 24 weeks; the blue rectangle indicates the time of delivery. There were two stillborns in each group. The remaining seven infants were challenged weekly with 20 TCID_50_ of SHIV1157ipd3N4 up to seven challenges until infection. If the infant remained uninfected after seven challenges, the dose was increased to 40 TCID_50_. Nx indicates necropsy. Illustrations of maternal and infant rhesus macaques are courtesy of Kathy West.

Rhesus monkey dams were primed with MVA 1086.C gp120 in the second trimester of pregnancy, followed by three subsequent gp120 i.m.-i.n. boosts (with STR8S-C and R848 adjuvants, respectively) during the third trimester of pregnancy, shortly after delivery, and at 3 weeks postpartum (Fig. 1). A group of control immunogen-vaccinated dams was included in this study for comparison. No significant adverse effects occurred as result of maternal immunization, and infant weight gain was similar to historical data. Starting 6 weeks after birth, to model late breast milk transmission, infants were orally challenged with repeated clade C Env simian-human immunodeficiency virus (SHIV). We then evaluated both maternal and infant immunological parameters to determine which factors predicted the risk of infant oral infection.

**FIG 2:**
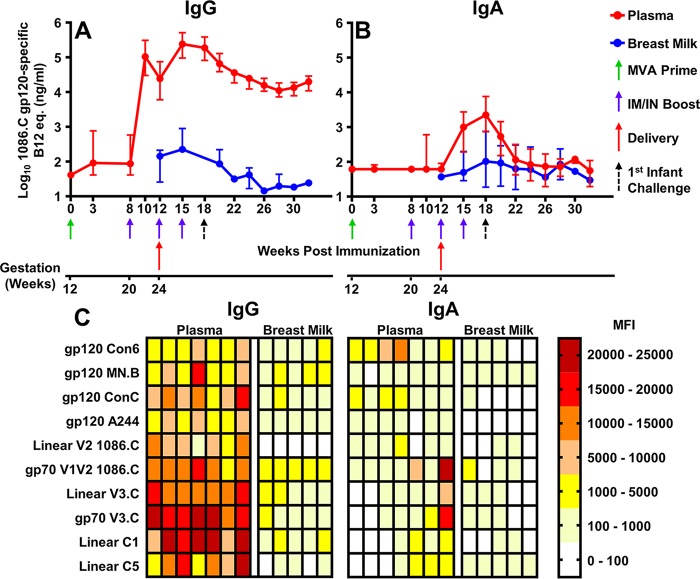
The maternal i.m. MVA gp120 prime–i.m.-i.n. gp120 boost regimen elicited high plasma gp120-specific IgG and milk IgA levels in rhesus monkey dams, including Abs against the critical gp120 epitopes V3, V1V2, and C1. Depicted are maternal vaccine-elicited 1086.C gp120-specific IgG (A) and IgA (B) responses in uninfected dams primed with a 1086.C-expressing MVA, followed by three successive i.m.-i.n. boosts during the second trimester of pregnancy and immediately postpartum. Data points represent median values for nine dams, and error bars represent ranges. Two dams delivered stillborns, and thus, responses in breast milk are unavailable for these animals. (C) Breadth and epitope specificity of responses to linear (denoted as linear) and conformational epitopes of gp120 at immunization week 18 after postpartum boosting. Maternal IgG (left panel) and IgA (right panel) are depicted for each individual dam, represented by a single column. Dams are displayed in the same order for IgG and IgA. There was not enough breast milk available from two dams to characterize their responses.

We first investigated the kinetics of the vaccine-elicited Ab responses in the pregnant dams (Fig. 2). Two weeks prior to delivery (2 weeks after the first protein boost), systemic 1086.C gp120-specific IgG levels in immunized dams were elevated (median, 106,692 ng/ml) (Fig. 2A). At the time of delivery (4 weeks after the first protein boost), these IgG levels in immunized dams had declined (median, 24,554 ng/ml), but they increased 3 weeks after delivery following the second i.m.-i.n. boost to peak levels in both plasma and breast milk (medians, 243,223 and 224 ng/ml, respectively) (Fig. 2A). The peak IgA response in plasma and milk occurred 6 weeks after delivery following the third i.m.-i.n. boost (medians, 2,239 and 103 ng/ml, respectively) (Fig. 2B), concurrent with the timing of the initial infant SHIV challenge. Vaccine-elicited Env-specific IgG levels in plasma and breast milk declined by 1 log between the peak response and 28 weeks postimmunization (16 weeks postdelivery), as did the Env-specific IgA level in plasma. Although the Env-specific IgA response in breast milk was small in magnitude, it was durable and persistent (Fig. 2A).

### Breadth of maternal vaccine-elicited plasma and milk IgG/IgA responses elicited by HIV-1 Env i.m.-i.n. immunization.

We evaluated the impact of the immunization regimen on both the breadth and epitope specificity of the vaccine-elicited maternal Ab responses (IgG/IgA) at 18 weeks postimmunization (3 weeks after the final protein boost), the time of the first oral SHIV exposure in infants, in both plasma and breast milk via binding Ab multiplex assay (BAMA) (Fig. 2C). Breadth was determined by measuring binding to a panel of consensus subtype B, C, AE, and group M gp120 proteins. All vaccinated dams developed plasma IgG binding responses to all of the gp120 clades tested in both plasma and breast milk. Similarly, systemic IgA binding to gp120 of all of the clades tested was detected in all but one of the immunized animals. However, there was no detectable binding of milk IgA to subtype C or AE gp120 in most of the animals. Epitope specificity of the vaccine-elicited Abs was measured by binding to linear and conformational epitopes of V1V2 and V3 and to linear epitopes of constant regions C1 and C5. The greatest magnitude systemic IgG binding was to the conformational V3 and linear C1 epitopes; however, strong binding to V1V2 and C5 was also observed. Interestingly, breast milk IgG binding to conformational V1V2 antigen (median mean fluorescence intensity [MFI], 2,487) was stronger than either conformational V3 or linear C1 responses (median MFI, 643 and 962, respectively). IgA epitope-specific responses varied in plasma and breast milk, yet the greatest magnitude systemic responses were against V1V2.

### Total and sIgA responses in milk elicited by HIV-1 Env i.m.-i.n. immunization.

**FIG 3:**
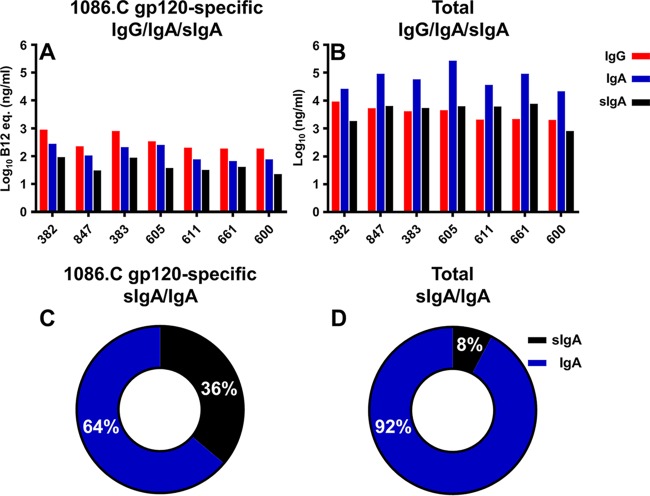
Breast milk vaccine-elicited gp120-specific IgA levels postpartum were similar in magnitude to those of IgG. Maternal levels of 1086.C gp120-specific (A) and total (B) IgG/IgA/sIgA levels in breast milk are shown for each of the vaccinated dams (*x* axis) after postpartum boosting. Env-specific sIgA constitutes 36% of the Env-specific IgA in breast milk (C), whereas the total sIgA constitutes a relatively smaller percentage (8%) of the total IgA in breast milk (D). Percentages represent averages for all of the dams tested. Breast milk was collected from dams 383 and 600 at week 15 postimmunization; milk was collected from all other dams at weeks 18 and 19.

The magnitude of the vaccine-elicited gp120-specific IgA response in breast milk following postpartum boosting was similar to that of IgG at matched time points (medians, 106 and 224 ng/ml; *P* = 0.06) (Fig. 3A), a finding similar to our previous observation in hormone-induced lactating rhesus monkeys (34). This comparable level of Env-specific milk IgG and IgA was achieved despite total levels of milk IgA being 1 log greater than the IgG level in postpartum rhesus dams (Fig. 3B), which mirrors human milk (27, 35). Interestingly, vaccine-elicited Env-specific sIgA levels in milk were lower than either Env-specific IgA or IgG levels in all of the animals (median, 38 ng/ml) (Fig. 3A). Yet, Env-specific sIgA comprised 36% of the Env-specific IgA in breast milk (Fig. 3C), whereas total sIgA constituted only 8% of the total IgA in breast milk (Fig. 3D). This overall small contribution of sIgA to the total IgA in milk is divergent from that measured in humans, where sIgA makes up an estimated 90% of the total milk IgA (36).

### Placental transfer of vaccine-elicited gp120-specific IgG at delivery is highly variable.

**FIG 4:**
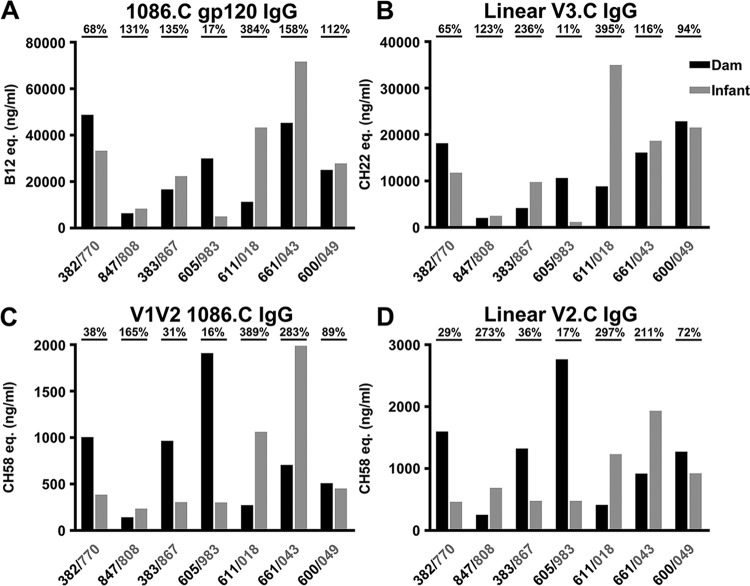
Variable placental transfer of vaccine-elicited gp120-specific IgG at delivery. The concentration in plasma of 1086.C Env-specific IgG (A), as well as epitope-specific IgG to V3 (B), V2 (D), and conformational V1V2 (C), for dams and infants at the delivery time point is shown. Delivery time point plasma was acquired 2 to 7 days after birth (median, 4). Dam data are black, and infant data are gray. Placental IgG transfer efficiency, measured as the ratio of infant to maternal antigen-specific IgG concentrations, is displayed for each dam-infant pair above the plot. Dam-infant pairs had higher levels of 1086.C gp120- and V3-specific than V1V2-specific IgG.

Considerable variation in IgG transfer efficiency, measured as the ratio of the infant to the maternal plasma antigen-specific IgG concentrations in the first week after delivery, was observed among the mother-infant pairs for each epitope-specific Ab population (Fig. 4). This variability in placental transfer ratios was not due to the range of infant sampling days (median, 4 days; range, 2 to 7 days), as maternal Ab levels were highest in the infant sampled at the greatest number of days after birth (7 days). Parity also did not correlate with placental transfer of the antigen-specific IgG tested. Differences in total transfer efficiencies across antigen-specific Ab populations were not remarkable. Interestingly, one mother-infant pair (605/983) had very inefficient placental IgG transfer across all epitopes (median, 16.5%). Notably, the median placental transfer efficiency across all antigens was <92% (range, 16.5 to 386.5%). While the placental IgG transfer efficiency was not well predicted by the magnitude of the plasma IgG response in the mother (*r* = −0.43, *P* = 0.35) (Fig. 4A), the magnitude of the maternal IgG response to linear and conformational V1V2 inversely correlated with the placental transfer efficiency of Abs directed against this epitope (*r* = −0.96, *P* = 0.003; *r* = −0.79, *P* = 0.048, respectively) (Fig. 4C and D). This low rate of placental transfer in some infants may be due to saturation of the neonatal Fc receptor (FcRn) by high levels of maternal IgG (37).

### Correlation between maternal and infant vaccine-elicited anti-HIV-1 Abs at delivery.

**FIG 5:**
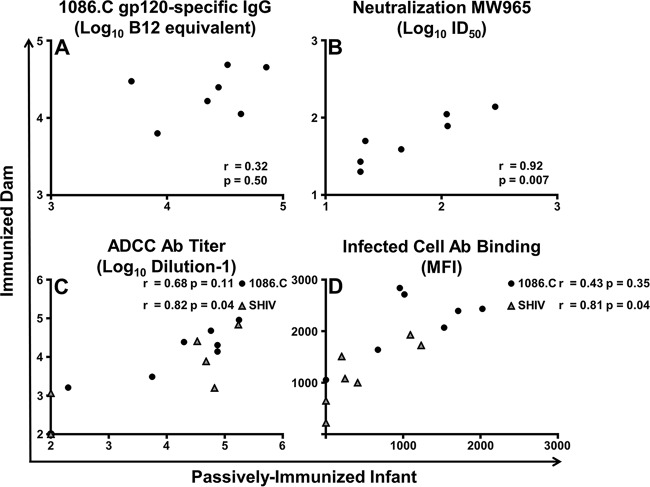
Correlation of maternal and infant vaccine-elicited binding and functional plasma Ab levels at delivery. Spearman’s correlation of 1086.C gp120-specific binding (A), neutralization titer (B), ADCC endpoint titer (C), and Ab binding to infected cells (D) is shown. For ADCC (C) and Ab binding to infected cells (D), data for 1086.C (black) and SHIV1157ipd3N4 (gray), the challenge virus, are shown. A strong correlation was observed between dam-infant pairs for neutralization of tier 1 clade C virus MW965, ADCC versus the SHIV challenge virus, and binding to cells infected with the SHIV challenge virus.

There was no evidence of a correlation between maternal and infant 1086.C gp120-specific IgG responses at delivery (*r* = 0.32, *P* = 0.50) (Fig. 5A). Nevertheless, a strong correlation between mother and infant neutralization titers versus tier 1 clade C virus MW965 was observed (*r* = 0.92, *P* = 0.007) (Fig. 5B). The mother and infant ADCC endpoint titers against cells coated with gp120 of SHIV1157ipd3N4, the challenge virus, were also strongly correlated (*r* = 0.82, *P* = 0.04) (Fig. 5C). However, the mother and infant plasma ADCC endpoint titers against gp120 of the vaccine-immunogen matched virus, 1086.C, did not exhibit a strong correlation (*r* = 0.68, *P* = 0.11). Likewise, there was a strong correlation between mother and infant plasma Ab binding to SHIV-infected cells (*r* = 0.81, *P* = 0.04) but no evidence of a correlation of Ab binding to 1086.C-infected cells (*r* = 0.43, *P* = 0.35) (Fig. 5D).

### Decline of passively transferred vaccine-elicited IgG in infant plasma.

**FIG 6:**
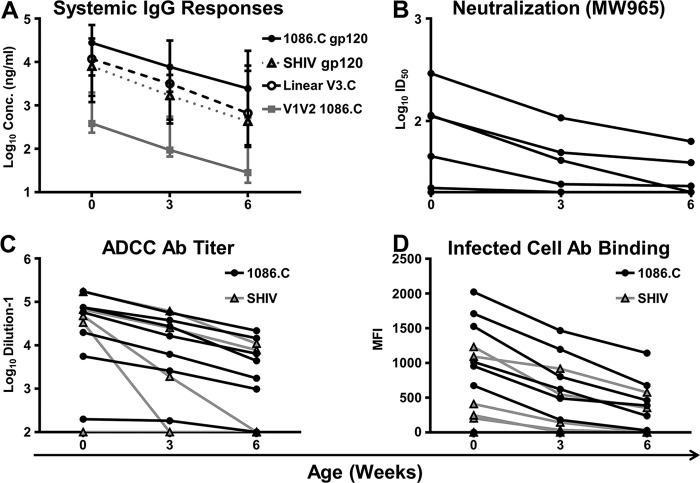
Vaccine-elicited Abs passively transferred to infants are capable of mediating low-level virus neutralization and ADCC. Infant plasma containing IgG Abs (A) against 1086.C, SHIV1157ipd3N4, linear (V3), and conformational V1V2 declined rapidly yet were initially able to mediate neutralization (B) versus tier 1 clade C virus MW965 and ADCC (C) against 1086.C and SHIV1157 gp120-coated target cells. Ab binding (D) to the surface of CEM.NKR_CCR5_ cells infected with either a 1086.C or an SHIV1157 infectious molecular clone was also observed at these early time points. In panel A, data points represent medians and error bars represent ranges. In panels B to D, lines represent individual infants.

Abs against the vaccine immunogen 1086.C gp120, as well as cross-reactive Abs to the challenge virus, SHIV1157ipd3N4, were present in infant plasma at birth (medians, 27,808 and 8,032 ng/ml, respectively) but declined steadily by the time of challenge (medians, 2,466 and 432 ng/ml; *P* = 0.02 and 0.02, respectively) (Fig. 6A). Vaccine-elicited Abs against key gp120 epitopes, V3 and V1V2, were also detected in infant plasma at birth (medians, 11,785 and 383 ng/ml, respectively). Similarly, these responses waned within the first 6 weeks of life and were 1 log lower at the time of challenge (medians, 663 and 28 ng/ml). Infant plasma was initially able to neutralize the tier 1 HIV-1 MW965 virus in 5/7 passively immunized infants at birth (median 50% inhibitory dilution [ID_50_], 45; range, <20 to 291) (Fig. 6B), but neutralization was detected in only 3/7 infants at the time of challenge (median ID_50_, 20; range, <20 to 63). Additionally, infant plasma demonstrated potent ADCC activity against 1086.C gp120-coated target cells both at birth (median endpoint titer, 57,803; range, 199 to 176,311) and at the time of the first challenge in 6/7 passively immunized infants (median endpoint titer, 4,413; range, <100 to 21,846) (Fig. 6C). We observed similar levels of ADCC activity directed against SHIV1157ipd3N4 gp120-coated target cells at delivery in 4/7 infants (median endpoint titer, 33,549; range, <100 to 171,895); however, at the time of challenge, only 2/7 infants had detectable ADCC responses. Similarly, we observed systemic Ab binding to cells infected with 1086.C in 6/7 infants at birth (median MFI, 1,016; range, 0 to 2,024) and the time of the first challenge (median MFI, 387; range, 0 to 1,142) (Fig. 6D). However, we only observed Ab binding to SHIV-infected cells in 5/7 passively immunized infants at birth (median MFI, 247; range, 0 to 1,232), which decreased to 2/7 infants at the time of the first challenge (MFI, 353 and 557).

### Similar risk of oral SHIV infection for maternal HIV-1 Env vaccine-elicited IgG passively immunized and control infants.

**FIG 7:**
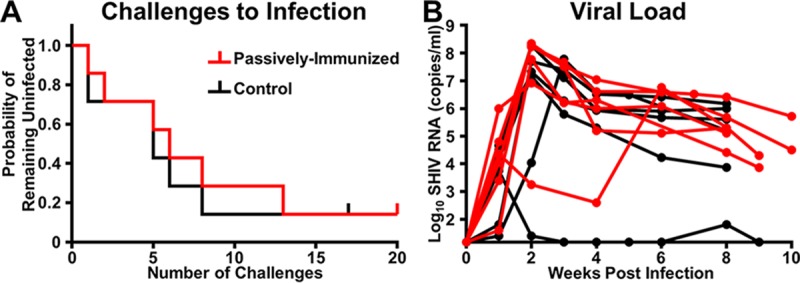
The distributions of the number of challenges to infection and median peak VL and set point were similar between treatment groups. The probability of remaining uninfected (A) was similar between passively immunized infants (red line) and control infants (black line) orally challenged with SHIV1157ipd3N4 (*P* = 0.72, exact log-rank test). The median peak VL (B) and set point at 8 weeks postinfection of infants in both groups were similar (*P* = 0.51 and 1.0, respectively; exact Wilcoxon rank sum test). The vertical notches in panel A represent the two uninfected monkeys. The lines in panel B represent individual infants that became infected.

Infants were orally challenged weekly with 20 50% tissue culture infective doses (TCID_50_) of SHIV1157ipd3N4 starting at 6 weeks of age until infection (Fig. 1). If infants remained uninfected after 7 challenges, the dose was increased to 40 TCID_50_, up to a maximum of 20 total challenges. At the conclusion of the study, 6/7 infants became infected in both the passively immunized and control groups (Fig. 7). The two uninfected infants received 20 and 17 challenges, respectively; all other infants became infected before 14 challenges. The Kaplan-Meier estimated median number of challenges to infection (6 and 5, respectively) and per challenge probability of infection (0.11 and 0.14, respectively) were similar in the passively immunized and control groups. There was no evidence of a difference between groups in the distribution of the number of challenges to infection (*P* = 0.72). There was no clinically significant difference in the peak and set point viral loads (VLs) (8 weeks postinfection, Table 1) between passively immunized and control infants (median peak VLs, 1.15 × 10^8^ and 3.55 × 10^7^ copies/ml, *P* = 0.51; median set point VLs, 1.65 × 10^5^ and 2.85 × 10^5^ copies/ml, *P* = 1.0, respectively). Interestingly, the number of challenges to infection for all infants inversely correlated with the set point VL (*r* = −0.67, *P* = 0.01) and did not correlate with the peak VL (*r* = −0.46, *P* = 0.10).

**TABLE 1:** Infant MHC class I and TRIM5α genotypes, number of challenges to infection, percentage of activated CD4^+^ T cells at the first challenge, and peak and set point VLs

Group and animal	Sex^a^	No. of reads mapped	MHC class I^b^				TRIM5 genotype^c^	Susceptibility score^d^	No. of challenges to infection	Wk 6 % activated CD4^+^ T cells	VL (no. of RNA copies/ml)	
			*Mamu-A* haplotype:		*Mamu-B* haplotype:						Peak	Wk 8 p.i.^e^
			1	2	1	2						
Passively immunized infants												
808	F	26,714	A008	A011	**B017a**	B043a	**TFP/CYP**	+2	Uninfected	0.91		<15
983	F	33,273	**A001**	A006	B048	B012a	TFP/TFP	0	13	2.93	170,000,000	32,000
770	F	25,501	**A001**	A004	B048	**B017a**	Q/Q	+1	8	0.39	59,000,000	26,000
049	F	33,176	A004	A012	B002	B043a	TFP/Q	0	6	0.98	8,200,000	130,000
018	F	25,560	A011	A019g1	B055	B013a	TFP/TFP	−1	5	3.64	220,000,000	200,000
043	F	26,592	A004	A123	B001a	B043c	TFP/TFP	−1	2	18.00	5,800,000	210,000
867	F	37,442	A012	A006	B001a	B001a	TFP/TFP	−1	1	6.17	190,000,000	2,600,000
Control infants												
048	M	32,804	**A001**	A002a	B048	B055	Q/CYP	0	Uninfected	1.08		<15
848	F	32,262	A004	A019g1	B047a	B047a	Q/Q	−1	8	4.57	15,000,000	7,400
849	F	33,090	A004	A004	B002	B012b	TFP/Q	0	6	3.83	21,000,000	410,000
839	M	31,520	A004	A003	B001a	B024a	TFP/TFP	−1	5	1.29	50,000,000	1,500,000
992	M	41,154	A004	A004	B002	B043a	TFP/Q	0	5	3.43	61,000,000	160,000
020	F	23,072	A004	A007	B012b	B077a	TFP/TFP	−1	1	9.91	190,000,000	1,000,000
040	F	64,939	A008	A123	B024a	B043c	TFP/TFP	−1	1	7.56	5,700	65

a F, female; M, male.

b The Mamu-A1*001, -B*008, and -B*017 alleles (bold) have been associated with a better SIV disease outcome.

c The TRIM5α TFP/CypA genotype (bold) confers resistance to SIVsmE660 (46), while the TFP/TFP, Q/Q, and Q/CypA genotypes are associated with increased susceptibility to SIVsmE660 infection.

d Protective MHC class I alleles and resistant TRIM5α alleles (Mamu-A1*001, -B*008, -B*017, and TFP/CypA) were assigned a value of 1. Genotypes associated with increased susceptibility or a worse disease outcome received a score of −1. All other alleles were assigned a score of 0. The susceptibility score represents the sum of all scores for an individual animal (e.g., animal 45770 has MHC class I scores of 0, 0, and +1 and a TRIM5α score of −1 for a score of +1).

e p.i., postinfection.

### Passively acquired binding and functional Ab responses in infants at the start of oral SHIV challenges do not correlate with the number of challenges to infection.

**FIG 8:**
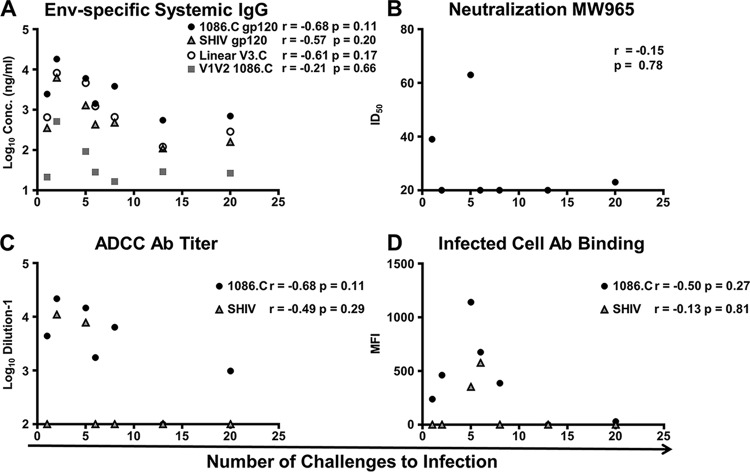
Lack of correlation between vaccine-elicited Ab responses in passively immunized infants and the number of challenges to infection. Envelope and epitope-specific Ab responses (A), neutralization (B), ADCC (C) and infected-cell binding (D) versus 1086.C and SHIV1157ipd3N4 measured at the time of the first challenge did not correlate with the number of challenges to infection. One infant remained uninfected at 20 challenges.

The levels of passively transferred Env-specific IgG in infants at 6 weeks of age did not correlate with the number of challenges to infection, potentially because of the variable transfer and rapid decline of the passively transferred vaccine-elicited IgG (Fig. 8A). Likewise, there was no evidence that the number of challenges to infection correlated with the infant plasma neutralization titer against MW965 (Fig. 8B) or the ADCC endpoint titer against 1086.C or SHIV1157ipd3N4 gp120-coated target cells (Fig. 8C). For example, infant 018, which became infected after five challenges, demonstrated both tier 1 neutralization and ADCC activity at 6 weeks of age (ID_50_, 63; ADCC endpoint titer, 7,810). Yet immunized infant 983, whose functional responses were undetectable at 6 weeks of age, resisted infection until 13 weeks of age. We further evaluated if binding of plasma Ab from passively immunized infants to 1086.C- or SHIV-infected cells at the time of challenge (Fig. 8D) correlated with the number of challenges to infection but similarly did not observe evidence of a correlation (*r* = −0.50, *P* = 0.27; *r* = −0.13, *P* = 0.81, respectively).

### Mucosal 1086.C gp120-specific IgG and IgA in passively immunized infants shortly after birth did not correlate with the number of challenges to infection.

Because of the potential role of mucosal Abs in preventing MTCT by breastfeeding (25), as well as previous studies that have demonstrated an association between salivary and fecal IgA levels with reduced acute viremia in adult rhesus macaques (38), we evaluated the levels of these potentially protective Abs in the present study. Indeed, we were able to detect both 1086.C gp120-specific maternal IgG and IgA Abs in saliva and stool samples from passively immunized infants (see Fig. S1 in the supplemental material). Additionally, we measured the ratio of specific activity of 1086.C gp120-specific IgG/IgA in both types of samples in the first week of life. Env-specific IgG activity in saliva was greater than that in stool (medians, 17.84 and 0.55 ng/μg, respectively) and 2 logs greater than Env-specific IgA activity in saliva (0.08 ng/μg) but we did not find evidence of a correlation with the peak or set point VL (*r* = −0.26, *P* = 0.66; *r* = 0.68, *P* = 0.11, respectively) or the number of challenges to infection (*r* = −0.75, *P* = 0.07). As seen with infant systemic IgG and functional Ab responses, mucosal IgG and IgA specific activity declined quickly at later time points.

10.1128/mSphere.00505-17.1 FIG S1  Lack of correlation between infant 1086.C gp120-specific IgG/IgA specific activity and challenges to infection. Infant Env-specific IgG and IgA levels in saliva (A and B) and stool (C and D) samples from passively immunized infants during the first week of life are shown. Notably, maternal Abs were detectable at mucosal sites. One infant remained uninfected at 20 challenges. Download FIG S1, TIF file, 0.5 MB. Copyright © 2018 Eudailey et al. 2018 Eudailey et al. This content is distributed under the terms of the Creative Commons Attribution 4.0 International license.

### Similar Ab responses in passively Env-immunized and control infants at 8 weeks postinfection.

We next sought to determine if the adaptive Ab responses to infection in the plasma of SHIV-infected monkeys developed distinctly in infants with and without passively acquired Env-specific IgG (Fig. S2). One infant in the control group, 040, exhibited transient viremia and no evidence of seroconversion and thus was excluded from this analysis. Eight weeks postinfection, both passively immunized and control infants developed similarly strong Ab binding responses to SHIV gp120 (*P* = 0.25) (Fig. S2A) and similar epitope specificity with comparable-magnitude responses against linear and conformational V3, as well as C5 (*P* = 0.33, 0.54, 0.33, respectively) (Fig. S2B). Interestingly, SHIV gp120 Ab binding in control infants showed evidence of a strong correlation with the set point VL at 8 weeks postinfection (*r* = 1, *P* = 0.02); however, this was not observed in passively immunized infants (*r* = −0.09, *P* = 0.92). Neither group mounted detectable responses against linear/conformational V1V2 or against C1 epitopes. Low-level plasma neutralization of MW965 was observed in four infants in each group (Fig. S2C), and the levels of neutralization in passively immunized and control infants did not differ (median ID_50_, 35 and 77, respectively; *P* = 0.79). Similar levels of ADCC against 1086.C gp120 (*P* = 0.13)-coated and SHIV1157ipd3N4 gp120 (*P* = 0.18)-coated target cells were also observed in the two groups (Fig. S2D).

10.1128/mSphere.00505-17.2 FIG S2  HIV Env-specific Ab responses and effector function at 8 weeks postinfection in passively immunized and control infants. (A) SHIV Env-specific IgG levels in plasma of passively immunized and control infants by week postinfection. Data points represent medians, and error bars represent ranges. (B) Epitope-specific responses to linear and conformational epitopes of gp120 of passively immunized (left side) and control (right side) infants at 8 weeks postinfection (necropsy). Individual infants (B) are represented by a single column. Neutralization (C) and ADCC (D) were similar in the two groups; horizontal bars represent median values. Infants that were uninfected or exhibited transient viremia without seroconversion were excluded. Download FIG S2, TIF file, 0.6 MB. Copyright © 2018 Eudailey et al. 2018 Eudailey et al. This content is distributed under the terms of the Creative Commons Attribution 4.0 International license.

### Direct correlation of activation status of infant systemic CD4^+^ T cells with the number of challenges to infection.

As we observed a wide range of numbers of challenges to infection (Table 1), we also investigated whether or not specific infant host factors were associated with the time to infection. We defined the major histocompatibility complex (MHC) class I and tripartite motif-containing protein 5 (TRIM5α) genotypes of the SHIV-challenged infants (Table 1). MHC alleles Mamu-A1*001, -B*008, and -B*017 have been associated with improved control of viral replication and a better disease outcome (39–44). Four out of five infants who were resistant to infection up to eight challenges all possessed one or more Mamu-A*001 or -B*017 alleles. Despite this finding, previous studies have associated protective MHC alleles with increased virologic control rather than protection against acquisition of SIV infection (45). The TRIM5α TFP/Cyp genotype confers resistance to low-dose simian immunodeficiency virus (SIV) SIVsmE660 (46), but not SIVmac239 and SIVmac251, infection (46, 47), while the genotypes TFP/TFP, Q/Q, and Q/Cyp are associated with increased susceptibility to SIVsmE660 infection (48, 49). Yet, none of these TRIM5 genotypes are likely to confer protection in this study, as the challenge virus has an SIVmac239 backbone (specifically, Gag) (46, 50). As expected, infants who required a greater number of challenges before infection showed no bias toward protective TRIM5 alleles, and conversely, infants who became infected after fewer challenges were not more likely to express alleles associated with greater susceptibility to infection.

**FIG 9:**
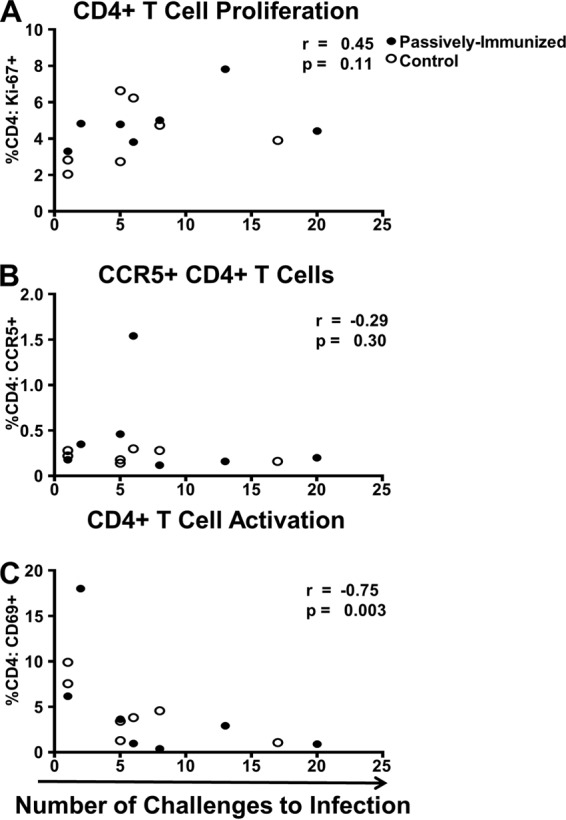
Infant systemic CD4^+^ T cell activation (CD69^+^) correlated with a reduced number of challenges to infection. CD4^+^ T cell proliferation (Ki-67^+^) (A) and CCR5^+^ expression (B), shown for all infants, did not show evidence of a correlation. Interestingly, CD4^+^ T cell activation (C) exhibited a strong correlation with a lower number of challenges to infection. Two infants remained uninfected at challenges 17 and 20. Passively immunized infants are represented by closed circles; control infants are represented by open circles.

Finally, there was no measurable difference in CD4^+^ T cell proliferation (Ki-67^+^), expression of the SHIV coreceptor CCR5, or activation (CD69^+^) between passively immunized and control infants (*P* = 0.38, 0.69, and 0.38). Thus, we examined all of the infants collectively (*n* = 14) but found no evidence that the percentage of CD4^+^ T cells in peripheral blood that were proliferating (intracellular Ki-67^+^ expression) or expressed CCR5 on their surface correlated with the number of challenges to infection (*r* = 0.45, *P* = 0.11; *r* = −0.29, *P* = 0.32, respectively) (Fig. 9A and B). Remarkably, a high percentage of infant circulating CD4^+^ T cells that expressed CD69 at the time of the first challenge strongly correlated with a low number of challenges to infection (*r* = −0.75, *P* = 0.003) (Fig. 9C). The proportion of variation in the number of challenges to infection explained by the percentage of CD4^+^ T cells that expressed CD69 was estimated to be 0.36 as determined by a discrete-time Cox model with percent CD4^+^ T cells specified quadratically.

## DISCUSSION

This study of maternal HIV-1 Env immunization of pregnant and lactating nonhuman primates (NHPs) demonstrates that an i.m. MVA gp120 prime–i.m.-i.n. gp120 boost immunization regimen is capable of eliciting both a robust systemic Env-specific IgG response and a durable milk IgA response, responses implicated in a reduced vertical transmission risk (23, 25). While these potentially protective Abs were transferred to the infant, the placentally transferred IgG waned quickly in infants and this passive IgG/IgA immunization did not impact protection against a heterologous tier 2 SHIV oral challenge starting at 6 weeks of age. A limitation of this model is that even though the infants were dam reared, virus inoculation occurred separately from breastfeeding; thus, Abs from breast milk may not have been present at a sufficient concentration in the oral cavity to prevent infection.

This model of maternal immunization followed by a subsequent infant oral SHIV challenge, mimicking HIV-1 exposure via breastfeeding, is the first to characterize the placental transfer of HIV-1 Env vaccine-elicited Abs in NHPs and investigate the efficacy of targeting breast milk immune responses. This model revealed that there is considerable variation in the efficiency of vaccine-elicited IgG transfer between rhesus monkey mothers and infants, yet the median transfer efficiency across Ab populations of distinct antigen specificity within the same host is similar. In addition to allowing us to measure the epitope specificity, breadth, and magnitude of breast milk Ab responses over time, this model also permitted us to study the relative composition of vaccine-elicited and total Ab isotypes in the milk of vaccinated dams. Surprisingly, using novel reagents to detect sIgA in the milk of rhesus monkeys (51), we found that the total sIgA constituted <10% of the total IgA in breast milk, in stark contrast to humans, where sIgA accounts for approximately 90% of the total milk IgA (36). This contrast in milk IgA isotype distribution may be an important distinction in human and NHP models that could impact the relative potency of protection; thus, additional studies should be performed to confirm this reduced sIgA-to-IgA ratio in rhesus macaque breast milk.

Despite induction of systemic and mucosal Abs that were passively transferred to the infant, there was no observed protection from infant oral SHIV acquisition in the passively immunized maternal vaccine group compared with the control group. Importantly, vaccine-elicited IgG in the dam that was placentally transferred to the infant rapidly declined in the first 6 weeks of life prior to the first challenge, as did the functional IgG responses (neutralization and ADCC), which were undetectable in more than half of the infants at the time of challenge. The average half-life of systemic IgG in rhesus macaques is 8.3 days (52); hence, maternal Abs transferred to infants prior to delivery may not have been present at levels sufficient for protection 6 weeks later. These findings, combined with the observation that maternal Ab responses did not peak until 6 to 8 weeks after delivery, demonstrate the need for additional boosting during pregnancy to reach maximal vaccine-elicited IgG transfer. These results highlight the importance of the timing of maternal vaccine administration during pregnancy. Specifically, to achieve optimal IgG transfer, the peak Ab levels should occur prior to delivery, which may require the administration of several vaccine doses in the second trimester and early in the third trimester of gestation, particularly in the case of a neoantigen. Active immunization of infants may also be required to elicit Env-specific IgG that persists at a level that confers protection, as passive immunization with a high concentration of neutralizing MAbs has been shown to mediate complete protection of neonatal rhesus macaques against an oral SHIV challenge (53). Additionally, a maternally administered HIV-1 vaccine could potentially enhance immune responses that could target the autologous maternal virus. Thus, a caveat of this model is that the dams were not SHIV infected, limiting our ability to measure the capacity of a maternal vaccine to raise levels of antiviral immunity to autologous virus and examine how this may impact transmission.

We evaluated the ability of the vaccine regimen to raise multiple potential humoral immune correlates of protection, including epitope-specific Ab binding and function. We further assessed genetic host factors previously associated with susceptibility to infection or disease outcome, including MHC class I and TRIM5α genotypes, which also did not seem to associate with the time to virus acquisition. Yet, interestingly, among all of the infants studied, there was a strong inverse correlation between the percentage of activated CD69^+^ CD4^+^ T cells in the peripheral blood at the time of the first challenge and the number of challenges to infection. This finding suggests that limiting the level of CD4^+^ T cell activation in breastfeeding infants may be of critical importance to further reducing postnatal virus transmission, which still occurs at a rate of 1 to 5% in HIV-1-exposed breastfeeding infants (54, 55). Infant monkeys in this study received solid food in addition to breast milk at the time of challenge, which has been associated with local and systemic immune stimulation that leads to increased intestinal mucosa permeability, which may facilitate viral entry (56). This recapitulates what often occurs in humans in resource-limited settings and may partially explain why mixed feeding has been associated with an increased risk of postnatal HIV-1 acquisition compared with exclusive breastfeeding (57).

In conclusion, while this maternal immunization strategy did not protect infants against repeated oral SHIV challenges, it importantly revealed the efficiency of passive transfer of vaccine-elicited IgG and milk IgA to the infant and provided key insights into the timing of maternal vaccine administration for optimal IgG transfer. Although placental transfer of vaccine-elicited IgG varied greatly between mother-infant pairs, perhaps earlier and repeated immunization boosts administered during the second and third trimesters of pregnancy could have mitigated this variability by achieving peak maternal IgG responses prior to delivery. With persistence of this passive immunity, protection would potentially be conferred on the infant during the breastfeeding period. One solution to ensure adequate passive IgG protection of the infant would be a subcutaneous dose of a broadly neutralizing Ab in the delivery room, which could potentially last throughout the early breastfeeding period, has been effective in preventing postnatal infection and clearing virus in perinatally infected infant monkeys (58), and is being studied in early-phase clinical trials (clinicaltrials.gov registration no. NCT02256631 and NCT03208231). Alternative prevention strategies are essential to further reducing the rate of MTCT, especially in areas where barriers to the implementation of ART exist. Consequently, additional studies are required to determine whether maternal or infant vaccine strategies can achieve this objective.

## MATERIALS AND METHODS

### Animal care and sample collection.

Adult female rhesus macaques were 5 to 16 years old, multiparous (average, four prior deliveries; range, one to nine), of Indian origin, and from the type D retrovirus-free, SIV-free, and simian T-lymphotropic virus type 1-free colony of the California National Primate Research Center (CNPRC), Davis, CA. Animals were maintained in accordance with the American Association for Accreditation of Laboratory Animal Care standards and the *Guide for the Care and Use of Laboratory Animals* (59). All protocols were reviewed and approved by the University of California at Davis Institutional Animal Care and Use Committee prior to the initiation of the study. Animals were housed indoors for an average of 28 (range, 10 to 64) months prior to time-mated breeding and kept indoors throughout the duration of this study. Pregnancy was confirmed and monitored regularly via ultrasound. When necessary, for sample collections and immunizations, animals were immobilized with ketamine HCl (Parke-Davis, Morris Plains, NJ) injected at 10 mg/kg of body weight. EDTA-anticoagulated blood was collected via peripheral venipuncture. Breast milk was collected manually from lactating dams under ketamine sedation. A dose of oxytocin (2 U in a volume of 0.1 ml) was given i.m. or subcutaneously 15 min prior to collection. Saliva and stool samples were collected at the birth time point, in the first week of life, and at weeks 3 (saliva) and 6 (stool) after birth. Saliva was not collected at week 6 (time of challenge) to avoid causing microabrasions that could facilitate viral entry into the oral cavity. Salivary secretions were collected with Weck-Cel sponges (Beaver Visitec. Waltham, MA) and immediately snap-frozen.

### Maternal immunization regimen.

Recombinant MVA expressing the 1086.C Env gp120-encoding gene and recombinant 1086.C Env gp120 glycoprotein were generated as previously described (32). Eighteen pregnant Indian-origin rhesus macaques (*Macaca mulatta*) housed at the CNPRC (Davis, CA) were randomly assigned to either the HIV-1 Env vaccine group (*n* = 9) or the control group (*n* = 9). Animals in the vaccine group were initially i.m. primed at 12 weeks of gestation with 10^8^ PFU of MVA expressing the HIV-1 Env 1086.C gp120-encoding gene, followed by three successive i.m.-i.n. 1086.C gp120 protein boosts at 20 weeks of gestation, at delivery, and at 3 weeks postpartum. The i.m. component of the combined boost consisted of 100 μg of HIV-1 Env 1086.C gp120 and 300 μl of STR8S-C adjuvant (squalene-containing STS base adjuvant plus R848 plus CpG oligodeoxynucleotides) (60) and was administered at a single injection site in the gluteus of anesthetized monkeys. The i.n. component consisted of 200 μg of HIV-1 Env 1086.C gp120 (100 μg per nostril) adjuvanted with the Toll-like receptor 7/8 agonist R848 (250 μg) and was administered to anesthetized monkeys placed on their backs in 25-μl doses at 30-s intervals for a total volume of 75 μl per nostril. Animals in the control group were primed i.m. with MVA without the gp120 insert and with respiratory syncytial virus fusion protein (RSV-F) DS-Cav1 (61) at 12 weeks of gestation, followed by i.m. RSV-F at 20 weeks of gestation. For the i.m. immunizations, 50 μg of RSV-F protein was adjuvanted with 500 μg of alum and delivered to the left and right biceps of anesthetized monkeys. The RSV vaccine used in this study is in a phase 1 clinical trial of healthy adults (clinicaltrials.gov registration no. NCT03049488) initiated in February 2017 and was previously tested in murine and NHP models. Blood was drawn directly before the initial MVA priming and following immunization at weeks 3, 8, 10, 12 (delivery time point), 15, and 18. All subsequent blood draws were conducted weekly up to 40 weeks postimmunization. Milk was collected starting at the delivery time point (12 weeks postimmunization, 2 to 7 days postdelivery) and following delivery at weeks 15 and 18 postimmunization. Milk was then collected on a weekly basis up to 32 weeks postimmunization. Two stillbirths occurred in both the treatment and control groups, but because of the small numbers, we cannot draw any definitive conclusions about the cause. Clinical and necropsy findings were consistent with dystocia due to large fetal size, with no presentation of congenital abnormalities, and the stillbirths were considered unrelated to immunizations.

### Preparation and titration of SHIV stock for infant oral challenge.

The SHIV1157ipd3N4 challenge virus stock was cultured and titers were determined as previously described (62, 63). In brief, concanavalin A-stimulated naive rhesus peripheral blood mononuclear cells (PBMCs) were infected with SHIV1157ipd3N4 (generously provided by Ruth Ruprecht) in the presence of human interleukin-2 (20 U/ml) and tumor necrosis factor alpha (10 ng/ml). Culture supernatants derived from donor animal PBMCs with the highest titers were pooled, and titers were determined in TZM-bl cells, yielding 18,275 TCID_50_/ml. To determine the appropriate virus titer for the study, four nursery-reared infants 11 to 14 weeks of age were sedated before receiving 1 ml of virus (800 TCID_50_) administered atraumatically into the oral cavity. Infants were challenged weekly until persistently infected, with a goal of infection of all of the infants after 10 inoculations. Infection was defined as two consecutive plasma RNA VL measurements of >15 copies/ml, with one of two measurements >150 copies/ml. The RNA VL was assessed weekly (Leidos Biomedical Research, Inc., Frederick, MD). All infants became infected after a single challenge; thus, the titer was determined to be too high. A second titration experiment was initiated with four infants at 6 weeks of age, starting with 10 TCID_50_/dose. After three challenges, two infants were infected. The dose was elevated to 50 TCID_50_ at the fourth challenge, after which the remaining two infants became infected. Twenty TCID_50_ was selected as the appropriate dose for this study to model infant exposure via breastfeeding.

Infants in this study were orally challenged weekly starting at 6 weeks of age with 20 TCID_50_ of SHIV1157ipd3N4 until persistently infected. This age was selected to model late breast milk transmission as would occur through prolonged breastfeeding, as opposed to neonatal transmission. Breast milk was not used to administer the virus, as it is known to contain multiple factors with antimicrobial and immunomodulatory properties that might have impacted the infectivity of the challenge virus and thus the risk of HIV-1 transmission via breastfeeding (64). These factors may have contributed to either anti-HIV-1 activity or inflammatory activity that may support HIV-1 transmission (64). The infectious dose was increased to 40 TCID_50_ on the eighth challenge if the infant remained uninfected, up to a maximum of 20 challenges. Blood was drawn at time zero (median, 4 [range, 2 to 7] days after birth) and week 3 and then weekly starting at the time of challenge (6 weeks of age) until 8 weeks postinfection.

The rate of natural transmission via breastfeeding is expected to be very low in infant macaques, and in the studies in which this was explored, it generally occurred quite late during breastfeeding (65–67). Additionally, the VL in breast milk over time and the frequency of viral shedding are highly variable in SIV infection of rhesus macaques (66). Oral inoculation with the challenge virus enabled more control over the timing of infection, the dose, and the ability to measure the number of challenges necessary to cause infection to determine the relative efficacy of the maternal HIV-1 vaccine.

### Blood and breast milk processing.

Plasma was separated from whole blood by centrifugation, and PBMCs were subsequently isolated by density gradient centrifugation with LSM lymphocyte separation medium (MP Biomedicals, Santa Ana, CA) as previously described (38, 68). Breast milk was separated into cellular, supernatant, and lipid fractions by centrifugation at 14,000 × *g* for 10 min at 4°C. The supernatant was collected and filtered via 0.22-μm Spin-X centrifugal filter tubes (Corning Life Sciences, Tewksbury, MA) by centrifugation at 9,000 rpm for 10 min at 4°C. Filtered breast milk supernatant was stored at −80°C for long-term storage or at 4°C for immediate use.

### Stool and saliva IgG/IgA detection by ELISA.

Saliva was eluted from sponges as described previously (69). For analysis of intestinal Abs, 2 to 3 g of freshly collected stool was added to 5 ml of sterile 1× phosphate-buffered saline (PBS) plus 0.5% bovine serum albumin supplemented with 50 μl of 100× protease inhibitor cocktail (Sigma-Aldrich, Corp., St. Louis, MO) and snap-frozen. Clarified fecal extracts were prepared as previously described (70) and concentrated to approximately 0.5 ml with an Amicon Ultra-4 50K centrifugal filter unit (Millipore, Billerica, MA).

1086.C gp120 IgG and IgA Ab levels in fecal extracts and saliva were measured by BAMA with gp120-conjugated Bio-Plex magnetic beads (Bio-Rad Laboratories, Inc., Hercules, CA) as previously described (71). Total IgG or IgA concentrations in secretions were measured by enzyme-linked immunosorbent assay (ELISA) with previously calibrated normal monkey serum as the reference standard (72). Anti-gp120 IgG or IgA concentrations measured in each secretion were divided by the total IgG or IgA concentration to obtain the specific activity (nanograms of IgG or IgA per microgram of total IgG/IgA). The specific activity was considered significant if it was greater than the mean activity measured in naive-infant control samples plus 3 standard deviations.

### Plasma IgG depletion.

Plasma samples were depleted of IgG prior to measurement of Env-specific and total IgA levels by ELISA and BAMA as previously described (27, 34). Plasma was first diluted 1:1 with 1× Tris-buffered saline (pH 7.5; Bio-Rad Laboratories, Inc., Hercules, CA) and then filtered via 0.22-μm Spin-X centrifugal filter tubes by centrifugation at 18,000 × *g* for 20 min at 4°C. Filtered plasma was added to a protein G Sepharose MultiTrap 96-well plate (GE Healthcare, Chicago, IL) packed with protein G resin and placed on an orbital shaker at 1,100 rpm for 1 h. The plate was centrifuged at 700 × *g* for 3 min at 20°C to elute the IgG-depleted plasma for use in IgA binding assays.

### BAMA.

The BAMA used to evaluate epitope specificity and breadth is similar to methods previously described (73). HIV-1 antigens (25 μg of each; Table S1) were covalently coupled to 5 × 10^6^ carboxylated fluorescent beads (Bio-Rad Laboratories, Inc., Hercules, CA), and then binding of plasma and milk IgG and IgA to the bead-coupled antigens was measured. Antigen-coupled beads and diluted plasma (IgG, 1:100; IgA, 1:5) or breast milk (IgG/IgA, 1:3) were incubated for 30 min at 20°C, and then IgG/IgA binding was detected with phycoerythrin-conjugated mouse anti-monkey IgG or goat anti-human IgA (Southern Biotech, Birmingham, AL) at 4 μg/ml. Beads were washed and acquired on a Bio-Plex 200 instrument (Bio-Rad Laboratories, Inc., Hercules, CA). Purified IgG from pooled plasma of HIV-1-vaccinated rhesus macaques (RIVIG) was used as a positive control. IgG and IgA binding was expressed as an MFI. All MFIs were adjusted by subtracting background values (MFI of coupled beads without sample). An HIV-1 Env-specific Ab response was considered positive if it had an MFI above the cutoff of 100. Consistency between assays was ensured by tracking the 50% effective concentration and maximum MFI of the positive control (RIVIG) by Levy-Jennings charts.

10.1128/mSphere.00505-17.3 TABLE S1  Amino acid sequences of antigens used in ELISAs and BAMAs in this study. Download TABLE S1, PDF file, 0.1 MB. Copyright © 2018 Eudailey et al. 2018 Eudailey et al. This content is distributed under the terms of the Creative Commons Attribution 4.0 International license.

### Env-specific and total IgG/IgA concentration measurement by ELISA.

High-binding 384-well plates (Corning Life Sciences, Tewksbury, MA) were coated at 45 ng/well with 1086.C gp120, SHIV1157ipd3N4 gp120, consensus V3.C peptide, V2 1086.C peptide, or scaffolded gp70 V1V2-1086.C (25) (Table S1) overnight at 4°C and then blocked with assay diluent (PBS containing 4% whey, 15% normal goat serum, and 0.5% Tween 20). Serially diluted plasma or breast milk was then added to the plate. IgG was detected with a horseradish peroxidase (HRP)-conjugated mouse anti-monkey IgG polyclonal Ab (Southern Biotech, Birmingham, AL). IgA Abs were detected with biotinylated mouse anti-rhesus IgA α-chain Ab 10F12 (NIH NHP Reagent Resource), followed by HRP-conjugated streptavidin (Thermo Fisher Scientific, Waltham, MA). ELISA plates were developed with SureBlue Reserve TMB substrate and stop solution (KPL, Gaithersburg, MD). Immediately after addition of the stop solution, plates were read at 450 nm, 0.1 s/well on a SpectraMax Plus 384 microplate reader (Molecular Devices, Sunnyvale, CA). Rhesus B12 IgG (b12R1) was used as a standard for IgG assays, and rhesus B12 IgA (b12RA1) was used as a standard for IgA assays (ranges, 5.7 × 10^−4^ to 100 and 5.7 × 10^−3^ to 1,000 ng/ml, respectively) (74). HIV-1 Env-specific IgG and IgA concentrations were calculated relative to the standard by using a five-parameter fit curve (SoftMax Pro 6.3; Molecular Devices, Sunnyvale, CA). The positivity cutoff was defined as the optical density (OD) of the lowest-concentration rhesus B12 standard that was greater than three times the average OD of blank wells.

To measure the total IgG/IgA concentration in milk by ELISA, high-binding 384-well plates (Corning Life Sciences, Tewksbury, MA) were coated overnight at 4°C with goat anti-monkey IgG or IgA (Rockland Immunochemicals, Inc., Limerick, PA) at 45 ng/well and then blocked with assay diluent for 1 h at 20°C. Diluted breast milk (1:50) was added to the plate, and it was incubated for 1 h at 20°C. Total IgG and IgA levels were detected and analyzed by assays based on the same methods and standards previously described for HIV-1 Env-specific IgG/IgA. The positivity cutoff was defined as the OD of the lowest-concentration rhesus B12 standard that was greater than two times the average OD of blank wells.

### Milk HIV-1 Env-specific and total sIgA concentration measurement by ELISA.

High-binding 384-well plates (Corning Life Sciences, Tewksbury, MA) were coated overnight at 4°C at 45 ng/well with 1086.C D7 gp120 (Env-specific) or goat anti-monkey IgA (total) (Rockland Immunochemicals, Inc., Limerick, PA). Plates were then blocked with assay diluent for 1 h at 20°C. Breast milk (undiluted for Env-specific IgA, 1:50 dilution for total IgA) was added to the plate, which was incubated for 1 h at 20°C. sIgA Abs were detected with an anti-secretory-chain IgA Ab, SC 9H7 CL3, a mouse-derived IgG2A kappa MAb that cross-reacts with the rhesus and human secretory components (SCs) but does not react with dimeric IgA (dIgA) alone (generously provided by Barton Haynes) (34, 51). This was followed by an HRP-conjugated anti-mouse IgG polyclonal Ab (Promega, Madison, WI). The assays were developed as previously described for HIV-1 Env-specific IgG/IgA. The standard used was B12 sIgA prepared by complexing B12 dIgA with rhesus SCs and incubating them overnight, which resulted in a 1:1 molar ratio of dIgA to SC. HIV-1 Env-specific sIgA concentrations were calculated relative to the standard by using a five-parameter fit curve (SoftMax Pro 6.3; Molecular Devices, Sunnyvale, CA). The positivity cutoff was defined as the OD of the lowest-concentration rhesus B12 sIgA standard that was greater than two times the average OD of blank wells.

### Neutralization assays.

Neutralization of tier 1 clade C infectious molecular clone MW965.LucR.T2A.ecto/293T by Abs in plasma was measured in TZM-bl cells (catalog no. 8129; NIH AIDS Reagent Program, Division of AIDS, NIAID, NIH; from John Kappes) via reduction of luciferase reporter gene expression after a single round of infection as previously described (63, 75, 76). Prior to screening, plasma was heated to 56°C for 30 min to inactivate complement. Luminescence was measured with a Victor X3 multilabel plate reader (PerkinElmer, Waltham, MA) at 1 s/well. The ID_50_ was calculated as the dilution that resulted in a 50% reduction in the number of relative luminescence units (RLU) compared to virus control wells. b12R1 was used as a positive control in each assay. The positivity cutoff for maternal plasma was three times the ID_50_ of the time zero preimmunization time point. As no preimmune sample existed for infants, because of passive maternal immunization, the positivity cutoff for infant plasma was three times the ID_50_ against murine leukemia virus (SVA.MLV) at birth. Cell surface expression of key markers is used to determine the authenticity of each new batch of TZM-bl cells, and cells are tested biannually for mycoplasma contamination.

### ADCC.

The GranToxiLux assay was used to detect ADCC activity in plasma directed against CEM.NKR_CCR5_ cells (catalog no. 4376; NIH AIDS Reagent Program, Division of AIDS, NIAID, NIH; from Alexandra Trkola) (77) coated with recombinant gp120 as previously described (78). Maternal and infant NHP plasma samples were tested for ADCC activity after 4-fold serial dilutions starting at 1:100. CEM.NKR_CCR5_ target cells were coated at 5 μg/ml with gp120 representing the vaccine immunogen, 1086.C D7 gp120 K160N, or the challenge virus, SHIV1157ipd3N4. Cryopreserved human PBMCs from an HIV-1-seronegative donor with the heterologous 158 F/V genotype for Fcγ receptor IIIa were used as the source of effector cells (79). ADCC endpoint titers were determined by interpolating the dilutions of plasma that intercept the positivity cutoff with GraphPad Prism v7 (GraphPad Software, Inc., La Jolla, CA). Cell surface expression of key markers is used to determine the authenticity of each new batch of CEM.NKR_CCR5_ cells.

### Plasma binding to the surface of HIV-1-infected cells.

Indirect surface staining was used to measure the ability of plasma samples to bind the HIV-1 envelope expressed on the infected-cell surface by methods similar to those previously described (80). CEM.NKR_CCR5_ cells were mock infected or infected with an HIV-1 infectious molecular clone (81) expressing the 1086.C or SHIV1157ipd3N4 envelope protein. The cells were incubated with a 1:100 dilution of plasma samples for 2 h at 37°C and then stained with Live/Dead Aqua Dead Cell Stain (Thermo, Fisher Scientific, Waltham, MA) to exclude dead cells from analysis. Cells were washed and then permeabilized with Cytofix/Cytoperm solution (BD Biosciences, San Jose, CA) prior to staining with fluorescein isothiocyanate (FITC)-conjugated goat anti-rhesus IgG (H+L) polyclonal antiserum (Southern Biotech, Birmingham, AL) and RD1-conjugated anti-p24 MAb KC57 (Beckman Coulter, Inc., Indianapolis, IN). Cells positive for plasma binding were defined as viable, p24/27 positive, and FITC positive. Final results are reported as the FITC MFI of the p24/27-positive events after subtraction of the background observed for the matched maternal prevaccination samples.

### Characterization of CD4^+^ T cell populations.

Suspensions of 1.5 × 10^6^ to 3.0 × 10^6^ PBMCs were stained for flow cytometry (for the Abs used, see Table S2). Paraformaldehyde-fixed samples were acquired on a BD LSR Fortessa instrument (BD Biosciences, San Jose, CA) with BD FACSDiva software and analyzed with FlowJo software version 10 (TreeStar, Inc., Ashland, OR). By the gating strategy used, lymphocytes were selected on the basis of the forward scatter area versus side scatter area profile and then single cells were selected by geometric gating, from which viable cells were selected. CD3^+^ CD4^+^ CD8^−^ T cells were further defined as CD69^+^ (activation), Ki-67^+^ (proliferation), or CCR5^+^ by utilizing Boolean gating on these parameters. Gating of CD4^+^ T-cell-specific populations was based on fluorescence-minus-one controls.

10.1128/mSphere.00505-17.4 TABLE S2  Abs used for flow cytometric phenotyping of CD4^+^ T cell populations in this study. Download TABLE S2, PDF file, 0.1 MB. Copyright © 2018 Eudailey et al. 2018 Eudailey et al. This content is distributed under the terms of the Creative Commons Attribution 4.0 International license.

### MHC class I and TRIM5α genotyping.

Genomic DNA (gDNA) was submitted to the Wisconsin National Primate Research Center (WNPRC), Madison, WI, to define the MHC class I and II and TRIM5α genotypes of SHIV-challenged infants as previously described (82). gDNA samples were used as templates for PCRs with a panel of primers that flank the highly polymorphic peptide binding domains encoded by exon 2 of class I (Mamu-A, -B, -I, and -E) and class II (Mamu-DRB, -DQA, -DQB, -DPA, and -DPB) loci. These PCR products were generated with a Fluidigm Access Array (Fluidigm, San Francisco, CA) that allows for multiplexing of all reactions in a single experiment. After cleanup and pooling, the amplicons were sequenced on an Illumina MiSeq instrument (Illumina, Inc., San Diego, CA) and the resulting sequence reads were mapped against a custom database of rhesus macaque class I and II sequences. Each sequence read is tagged with a unique set of barcodes and thus can be traced back to the infant from which it originated. In total, nearly 1.2 million sequence reads were identified with an average of 30,679 reads/infant. PCR sequence-specific primer assays were performed to detect the TRIM5-Q and TRIM5-TFP alleles as previously described (46). Likewise, the TRIM5-CypA allele was detected by NsiI restriction digestion (83).

### Statistical methods.

Hypothesis tests from planned analyses were not adjusted for multiple comparisons, and results were considered statistically significant for *P* values of <0.05. Planned analyses included the following: change in Env-specific IgA response in breast milk over time, vaccine-elicited gp120-specific IgA in breast milk following postpartum boosting versus IgG, placental transfer of vaccine-elicited gp120-specific IgG correlations, maternal versus infant vaccine-elicited functional anti-HIV-1 Ab correlations, change in infant Ab responses over time, and challenge study analyses. Hypothesis tests for exploratory analyses were considered significant if the *P* value was less than the threshold defined by a false-discovery rate of 0.05; by this criterion, none of the *P* values reported for exploratory analyses were significant. Exploratory analyses included the following: tests of association with the number of challenges to infection excepting comparison to set point and peak VLs, set point and peak VLs compared to Env-specific IgG activity in saliva, associations with V3 and C5 binding responses, and comparison of CD4 T cell proliferation (Ki-67^+^), CCR5 expression, and activation (CD69^+^). Comparisons of immunization groups were done with Wilcoxon rank sum tests, and comparisons within monkeys were done with Wilcoxon signed-rank tests. Correlations were calculated with Spearman’s rank correlation coefficient. For correlation calculations that included the number of challenges to infection as a variable, monkeys that were not infected by the end of the challenge study were assigned the highest rank for the number of challenges to infection. The number of infections per SHIV challenge was used to estimate the per-challenge probability of infection. Kaplan-Meier curves were created to estimate the distributions of the number of challenges to infection for both passively immunized and control groups. The distributions of the number of challenges to infection were compared between the passively immunized and control groups by using a log-rank test; monkeys not infected by the end of the challenge study were treated as right censored (84–86). All hypothesis testing utilized exact *P* values and was two tailed. Statistical analysis was performed with GraphPad Prism v7 (GraphPad Software, Inc., La Jolla, CA) and R statistical software v3.3.0 (R Project for Statistical Computing, Vienna, Austria).

### Data availability.

In accordance with the NIH Public Access Policy (NOT-OD-08-033), all investigators will submit an electronic version of their final peer-reviewed work to the National Library of Medicine PubMed Central to be made publicly available no later than 12 months after the official date of publication. In addition, unpublished data and the associated information will be made available via Excel databases or SAS files (as appropriate for the data) to others after publication under a data-sharing plan that provides that (i) data can only be used for research purposes, (ii) data must be secured by using appropriate computer technology, (iii) data must be destroyed or returned after analyses are completed, (iv) data may not be transferred to a third party, and (v) the source of the data set must be acknowledged in any publications or public presentations. Upon request, we will make the raw data, as well as tables and graphs generated from the data, available to the scientific community.
