# Supplementary figures and images for: Maternal HIV-1 Env Vaccination for Systemic and Breast Milk Immunity To Prevent Oral SHIV Acquisition in Infant Macaques
Source: mSphere. 2018 Jan 10;3(1):e00505-17. doi: 10.1128/mSphere.00505-17 (PMC5760748; doi:10.1128/mSphere.00505-17)

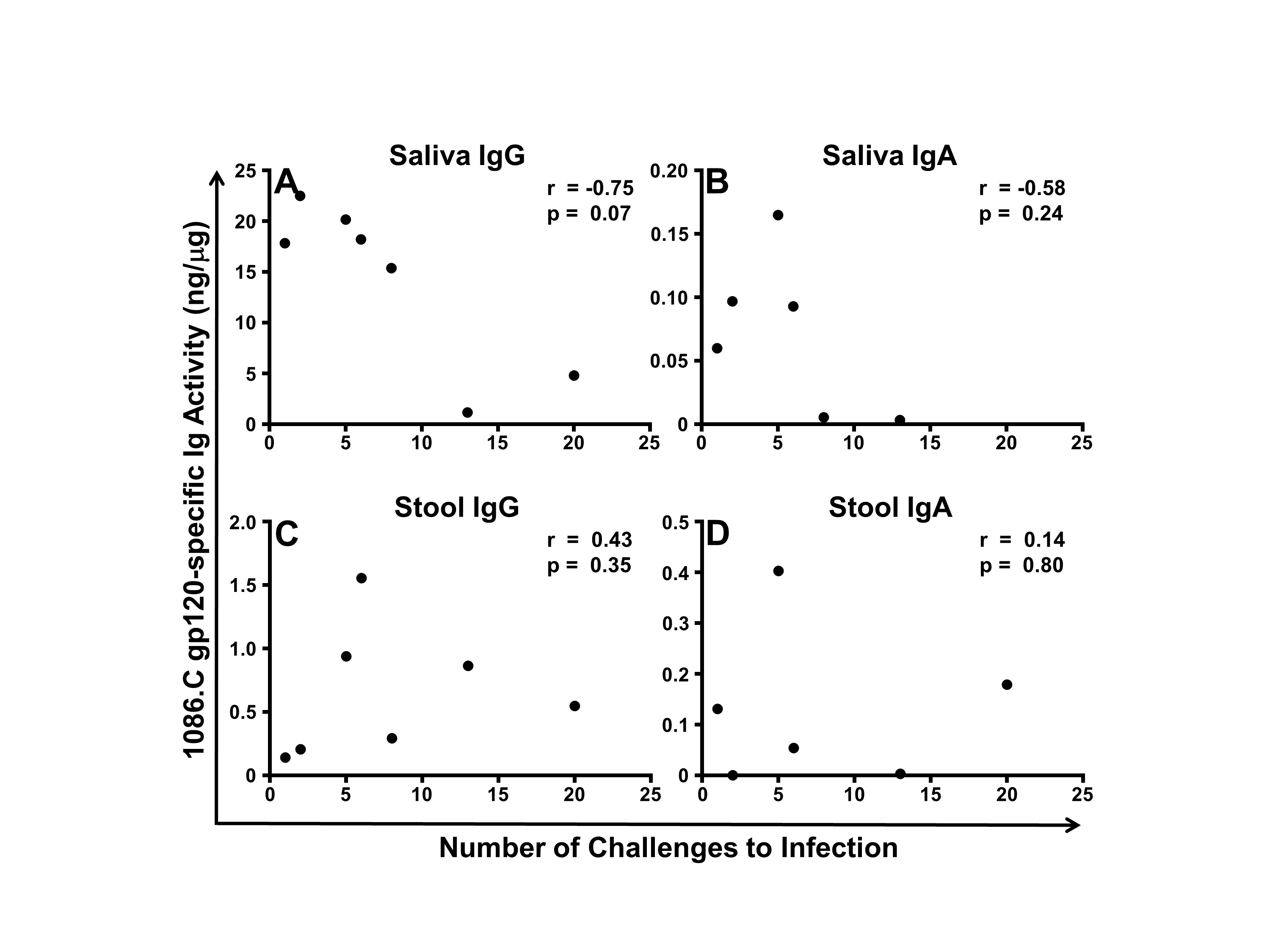

Supplement: FIG S1 [file sph001182446sf1.tif]

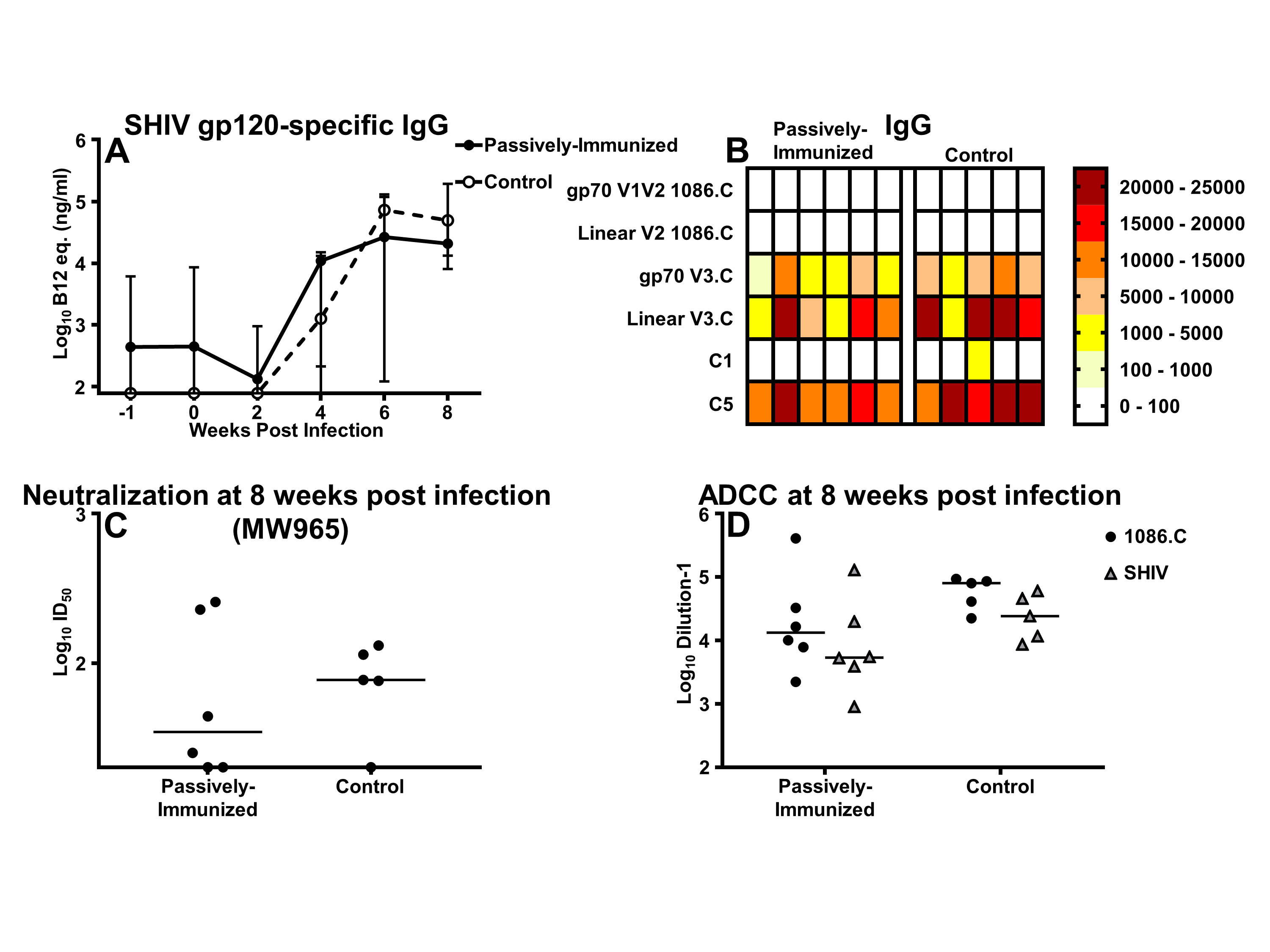

Supplement: FIG S2 [file sph001182446sf2.tif]
