# Supplementary material for: Maternal HIV-1 Env Vaccination for Systemic and Breast Milk Immunity To Prevent Oral SHIV Acquisition in Infant Macaques
Source: mSphere. 2018 Jan 10;3(1):e00505-17. doi: 10.1128/mSphere.00505-17 (PMC5760748; doi:10.1128/mSphere.00505-17)
Supplement: TABLE S1 [file sph001182446st1.pdf]

Supplemental Table 1. Amino acid sequences for antigens used in ELISA and BAMA assays.

Assay	Protein	Peptide	Sequence
ELISA	1086D7 gp120K160N/293F		SVPVWKEAKTTLFCASDAKAYEKEVHNVWATHACVPTDPNPQEMVLANVTENFNMWKNDMVEQMHEDIISLWDESLKPCVKLTPLCVTLNCTNVKGNESD<br>TSEVMKNCSFNATTELKDKKHKVHALFYKLDVVPLNGNSSSSGEYRLINCNTSAITQACPKVSFDPIPLHYCAPAGFAILKCNNKTFNGTGPCRNVSTVQ<br>CTHGIKPVVSTQLLLNGSLAEEEIIIRSENLTNNAKTIIVHLNESVNIVCTRPNNNTRKSIRIGPGQTFYATGDIIGNIRQAHCNINESKWNNTLQKVGE<br>ELAKHFPSKTIKFEPSSGGDLEITTHSFNCRGEFFYCNTSDLFNGTYRNGTYNHTGRSSNGTITLQCKIKQIINMWQEVGRAIYAPPIEGEITCNSNITG<br>LLLLRDGGQSNETNDTETFRPGGGDMRDNWRSELYKYKVVEIKPLGVAPTEAKRRVVEREKR
	SHIV1157ipd3N4 D11 gp120		VPVWKEAKTTLFCASNAKAYEKEVHNIWATHACVPTDPNPQEIVLGNVTENFNMWKNDMVDQMHEDIISLWDQSLKPCVKLTSLCVTLKCSNFTGKSNVT<br>YKGDMEVKNCSFNVTTEIRDKKQKVYALFYRLDITPLDDNSSEYILINCNSSTITQACPKVNFDPIPIHYCAPAGYAILKCNNKTFNGTGPCHNVSTVQC<br>THGIKPVVSTQLLLNGSLAEGEIIIRSENLTDNVKTIIVHFNESVEITCTRPNNNTRKSISIGPGQAIYATGDIIGDIRQAHCNISKENWNKTLQWVRGK<br>LKEHFPNKTIVFKPSSGGDLEITTHSFNCRGEFFYCNTSKLFNSTDNSTHMGTENNTIITIPCRIKQIINMWQEVGRAMYAPPIEGNITCKSNITGLLLV<br>RDGGWDNSTNDTETFRPGGGDMRDNWRSELYKYKVVEVKPLGIAPTKAKERVVEREKE
		(Linear) Bio-V3. C	KKKNNTRKSIRIGPGQTFYATGDIIGDIRQAHC
		(linear) Bio-V2 1086.C	KKKTELKDKKHKVHALFYKLDVVP
		gp70 V1V2 1086.C	CTNVKGNESDTSEVMKNCSFKATTELKDKKHKVHALFYKLDVVPLNGNSSSSGEYRLINC
BAMA	Con 6 gp120		AENLWVTVYYGVPVWKEANTTLFCASDAKAYDTEVHNVWATHACVPTDPNPQEIVLENVTENFNMWKNNMVEQMHEDIISLWDQSLKPCVKLTPLCVTLN<br>CTNVRNVSSNGTETDNEEIKNCSFNITTELRDKKQKVYALFYRLDVVPIDDKNSSEISGKNSSEYYRLINCNTSAITQACPKVSFEPIPIHYCAPAGFAI<br>LKCNDKKFNGTGPCKNVSTVQCTHGIKPVVSTQLLLNGSLAEEEIIIRSENITNNAKTIIVQLNESVEINCTRPNNNTRKSIHIGPGQAFYATGEIIGDI<br>RQAHCNISRTKWNKTLQQVAKKLREHFNNKTIIFKPSSGGDLEITTHSFNCGGEFFYCNTSGLFNSTWMFNGTYMFNGTKDNSETITLPCRIKQIINMWQ<br>GVGQAMYAPPIEGKITCKSNITGLLLTRDGGNNSNKNKTETFRPGGGDMRDNWRSELYKYKVVKIEPLGVAPTKAKRRVVEREKR
	MN gp120 gD neg/293F/mon		TEKLWVTVYYGVPVWKEATTTLFCASDAKAYDTEAHNVWATHACVPTDPNPQEVELVNVTENFNMWKNNMVEQMHEDIISLWDQSLKPCVKLTPLCVTLN<br>CTDLRNTTNTNNSTDNNNSKSEGTIKGGEMKNCSFNITTSIGDKMQKEYALLYKLDIEPIDNDSTSYRLISCNTSVITQACPKISFEPIPIHYCAPAGFA<br>ILKCNDKKFSGKGSCKNVSTVQCTHGIRPVVSTQLLLNGSLAEEEVVIRSEDFTDNAKTIIVHLKESVQINCTRPNYNKRKRIHIGPGRAFYTTKNIKGT<br>IRQAHCIISRAKWNDTLRQIVSKLKEQFKNKTIVFNPSSGGDPEIVMHSFNCGGEFFYCNTSPLFNSIWNGNNTWNNTTGSNNNITLQCKIKQIINMWQK<br>VGKAMYAPPIEGQIRCSSNITGLLLTRDGGEDTDTNDTEIFRPGGGDMRDNWRSELYKYKVVTIEPLGVAPTKAKRRVVQREKR
	ConC gp120 WT		NLWVTVYYGVPVWKEAKTTLFCASDAKAYEKEVHNVWATHACVPTDPNPQEMVLENVTENFNMWKNDMVDQMHEDIISLWDQSLKPCVKLTPLCVTLNCR<br>NVTNATNNTYNEEIKNCSFNITTELRDKKKKVYALFYRLDIVPLNENSSEYRLINCNTSAITQACPKVSFDPIPIHYCAPAGYAILKCNNKTFNGTGPCN<br>NVSTVQCTHGIKPVVSTQLLLNGSLAEEEIIIRSENLTNNAKTIIVHLNESVEIVCTRPNNNTRKSIRIGPGQTFYATGDIIGDIRQAHCNISEDKWNKT<br>LQRVSKKLKEHFPNKTIKFEPSSGGDLEITTHSFNCRGEFFYCNTSKLFNSTYNNNTNSNSTITLPCRIKQIINMWQEVGRAMYAPPIAGNITCKSNITG<br>LLLTRDGGKKNTTEIFRPGGGDMRDNWRSELYKYKVVEIKPLGVAPTKAKRRVVEREKR
	A244 gp120 gDneg/293T		SDNLWVTVYYGVPVWKEADTTLFCASDAKAHETEVHNVWATHACVPTDPNPQEIDLENVTENFNMWKNNMVEQMQEDVISLWDQSLKPCVKLTPPCVTLH<br>CTNANLTKANLTNVNNRTNVSNIIGNITDEVRNCSFNMTTELRDKKQKVHALFYKLDIVPIEDNNDSSEYRLINCNTSVIKQPCPKISFDPIPIHYCTPA<br>GYAILKCNDKNFNGTGPCKNVSSVQCTHGIKPVVSTQLLLNGSLAEEEIIIRSENLTNNAKTIIVHLNKSVVINCTRPSNNTRTSITIGPGQVFYRTGDI<br>IGDIRKAYCEINGTEWNKALKQVTEKLKEHFNNKPIIFQPPSGGDLEITMHHFNCRGEFFYCNTTRLFNNTCIANGTIEGCNGNITLPCKIKQIINMWQG<br>AGQAMYAPPISGTINCVSNITGILLTRDGGATNNTNNETFRPGGGNIKDNWRNELYKYKVVQIEPLGVAPTRAKRRVVEREKR
		(linear) Bio-V2 1086.C	KKKTELKDKKHKVHALFYKLDVVP
		gp70 V1V2 1086.C	CTNVKGNESDTSEVMKNCSFKATTELKDKKHKVHALFYKLDVVPLNGNSSSSGEYRLINC
		(Linear) Bio-V3. C	KKKNNTRKSIRIGPGQTFYATGDIIGDIRQAHC
		gp70 ConC V3 tags	CTRPNNNTRKSIRIGPGQTFYATGDIIGDIRQAHC
		(Linear) C1 Biotin	KKKMQEDVISLWDQSLKPCVKLTPLCV
		inear C5) Bio_RV144_C5.2	KKKSELYKYKVVEIKPLGIAPTKAKRRVVEREKRAV
