# Supplementary material for: Maternal HIV-1 Env Vaccination for Systemic and Breast Milk Immunity To Prevent Oral SHIV Acquisition in Infant Macaques
Source: mSphere. 2018 Jan 10;3(1):e00505-17. doi: 10.1128/mSphere.00505-17 (PMC5760748; doi:10.1128/mSphere.00505-17)
Supplement: TABLE S2 [file sph001182446st2.pdf]

**Supplemental Table 2. Abs used for flow cytometric phenotyping of CD4+ T cell populations.**

**CD4+ T Cell Panel**

| Marker | Surface/Intracellular Staining[a] | Fluorophore | Clone | Vendor | Catalog No. |
|---|---|---|---|---|---|
| Live/Dead | | Yellow | N/A | Invitrogen | L34959 |
| CD3 | S/IC | BV421 | SP34-2 | BD Biosciences | 562877 |
| CD4 | S | PerCP-Cy5.5 | L200 | BD Biosciences | 552838 |
| CD8 | S | Alexa Fluor 700 | RPA-T8 | BD Biosciences | 557945 |
| CD69 | S | PE-Cy7 | FN50 | BD Biosciences | 557745 |
| CCR5 | S | PE | 3A9 | BD Biosciences | 550632 |
| Ki-67 | IC | FITC | B56 | BD Biosciences | 556026 |
| TNF-α | IC | BV560 | MAb11 | BD Biosciences | 563418 |

[a]S indicates surface staining, IC indicates intracellular staining.
